# L-Arginine Supplementation Did Not Impact the Rapid Recovery of Cardiovascular and Autonomic Function Following Exercise in Physically Active Healthy Males: A Triple-Blind Randomised Placebo-Controlled Crossover Trial

**DOI:** 10.3390/nu16234067

**Published:** 2024-11-27

**Authors:** Andrey Alves Porto, Luana Almeida Gonzaga, Felipe Ribeiro, Camila Marcondes de Oliveira, Luiz Carlos Marques Vanderlei, Vitor Engrácia Valenti

**Affiliations:** 1 Postgraduate Program in Movement Sciences, Sao Paulo State University (UNESP), Presidente Prudente 19060-900, SP, Brazil; luana.gonzaga@unesp.br (L.A.G.); felipe.ribeiro1@unesp.br (F.R.); lcm.vanderlei@unesp.br (L.C.M.V.); vitor.valenti@unesp.br (V.E.V.); 2 Systematic Reviews Center for Cardiovascular and Metabolic Health, Sao Paulo State University (UNESP), Marilia 17525-900, SP, Brazil; camilamaao@unesp.br

**Keywords:** arginine, exercise, autonomic nervous system, nitric oxide and post-exercise recovery techniques

## Abstract

Background and Aims: Post-exercise recovery strategies include massage, low-intensity active exercise, thermal contrast, hydration, and nutritional and herbal approaches. These strategies aim to accelerate recovery, enhance performance, and optimise the physical training process. L-arginine (L-ARG) is the physiological precursor of nitric oxide (NO), a crucial mediator of vasodilation and the inhibition of platelet aggregation. A previous study reported that L-ARG supplementation could significantly reduce the systolic blood pressure (SBP) and diastolic blood pressure (DBP). This study aimed to investigate the effects of L-ARG on autonomic and cardiovascular recovery immediately following submaximal exercise. Methods and Results: Thirty-two healthy individuals were subjected to two experimental protocols. The first protocol included 60 min of rest, a treadmill warm-up, and load increments until reaching 80% of their maximum HR. Before this protocol, the subjects consumed 3 g of starch (placebo protocol). The second protocol was identical, but the subjects consumed 3 g of L-ARG. Heart rate recovery (HRR), heart rate variability (HRV), and blood pressure (BP) responses were assessed. No significant differences in HRR were found (*p* = 0.944) regarding the root mean square of successive differences in the RR interval (RMSSD30) of HRV (*p* = 0.562) or in the BP responses (mean arterial pressure (MAP), *p* = 0.687; pulse pressure (PP), *p* = 0.929) between the protocols. Conclusions: L-ARG supplementation did not significantly alter immediate post-exercise autonomic recovery in healthy males.

## 1. Introduction

Studying strategies that optimise post-exercise recovery is essential to prevent injuries, improve workout performance, and reduce the risk of adverse cardiac events. Effective recovery methods contribute to health and well-being by ensuring a quick and efficient recovery, minimising physical stress, and improving the ability to perform physical activities safely [1].

Exercise acts as a stimulus to the body, eliciting a response from the autonomic nervous system (ANS). During physical exertion, there is a reduction in parasympathetic activity and an augmentation in sympathetic activity to meet the metabolic demands. Following the cessation of exercise, autonomic recovery is characterised by a significant decrease in heart rate (HR) [2]. Thus, heart rate recovery (HRR) can be a non-invasive tool to evaluate the function of the ANS [3] and HRV [4,5].

In this context, and to monitor the subjects’ health status, the heart rate (HR) variability (HRV)—a measure of heartbeat oscillation that reflects autonomic nervous system function [6,7]—has been shown to provide valuable clinical insights into the health status [8,9,10,11]. HRV has been investigated for many years, with growing interest in understanding its mechanisms and clinical applications in various diseases. The clinical significance of HRV was first recognised when it demonstrated its value in monitoring foetal distress. Later, an association was found between reduced HRV and an elevated risk of mortality following acute myocardial infarction, supporting the use of HRV as a strong and independent predictor of mortality after acute myocardial infarction [12]. Therefore, using HRV to assess quality of life enhances our understanding of patients’ health.

The pioneering study by Cole et al. (2000) [5] analysed the HRR after a submaximal exercise test as a predictor of mortality in healthy adults. In a cohort of 5234 participants without cardiovascular disease, abnormal HRR (defined as ≤42 bpm within the first two minutes of recovery) was associated with a higher rate of death. During 12 years of follow-up, abnormal HRR remained a significant predictor of mortality, even after adjusting for various risk factors, with an adjusted relative risk of 1.55 (CI, 1.22 to 1.98), thereby highlighting the clinical importance of HRR in submaximal exercise testing.

Post-exercise recovery strategies include techniques such as massage, low-intensity active exercise, thermal contrast (hot–cold) [1], and hydration [13], as well as nutritional and herbal approaches [14]. These strategies aim to accelerate recovery, enhance performance, and optimise the physical training process [1,4]. Dietary supplements, especially those containing L-ARG, are frequently recommended to help boost energy levels and reduce muscle fatigue [15,16]. L-ARG is the physiological precursor of NO, a crucial mediator of vasodilation and the inhibition of platelet aggregation through the enhancement of cyclic guanosine monophosphate (GMP) formation [17].

Shiraseb et al. (2022) [18] reported that L-ARG supplementation can significantly reduce the SBP and DBP. Their meta-analysis, which included 22 studies, revealed a weighted mean reduction of 6.40 mmHg in the SBP and 2.64 mmHg in the DBP. Subgroup analyses indicated that L-arginine supplementation consistently lowered the blood pressure, regardless of the baseline blood pressure category (normal, elevated, or hypertension), body mass index (normal, overweight, or obese), or health status.

Furthermore, NO is associated with more efficient autonomic modulation in healthy adults. In the study by Chowdhary et al. (2000) [19], the authors investigated the impact of NO on baroreflex HR control. Their study involved 26 male volunteers and assessed HRV and baroreflex sensitivity during endogenous NO production inhibition with L-NG-monomethylarginine (L-NMMA) and exogenous NO administration with sodium nitroprusside. The results demonstrated that NO enhances cardiac vagal control, as evidenced by the preservation of high-frequency HRV indices during NO administration.

Moreover, there is a body of research examining the effects of L-arginine on cardiovascular and autonomic responses, which has yielded a range of findings. For instance, a study by Streeter et al. in 2019 [20] specifically evaluated L-arginine’s influence on recovery following resistance exercise, concluding that it did not significantly improve recovery metrics. This study highlights a particular context in which L-arginine supplementation might not provide the expected benefits. In addition, other studies have explored L-arginine’s broader impact on athletic performance, primarily due to its crucial role in the synthesis of nitric oxide. Nitric oxide plays a vital role in vasodilation, potentially enhancing blood flow and oxygen delivery to the muscles, which is critical during and after physical exertion.

Another study [21] examined the effects of citrulline malate (CM) supplementation on CrossFit^®^ performance and the cardiovascular response. Although CM did not significantly improve the workout rounds completed, it increased the time in higher-heart-rate zones, potentially enhancing the aerobic capacity. No significant impact was found on the post-exercise recovery time. Additionally, others [22] found that pre-exercise L-citrulline supplementation in Yili speed-racing horses improved their performance, reduced their post-race lactate levels, accelerated their recovery, and enhanced their antioxidant capacity. Supplementation also increased plasma citrulline, arginine, and other beneficial markers, suggesting positive physiological and biochemical effects during high-intensity exercise.

Although the academic literature frequently focuses on the effects of L-ARG supplementation on performance and exercise outcomes, this study aimed to investigate the impact of L-ARG on autonomic and cardiovascular recovery immediately following submaximal exercise. This study provides insights into the potential reduction in acute risks post-exercise. It explores interventions that could guide healthcare professionals on the use of L-ARG in healthy individuals and inform future research exploring its effects on various health conditions across different populations.

## 2. Methods

### 2.1. Ethical Aspects and Population

The Research Ethics Committee of the Faculty of Philosophy and Sciences at Universidade Estadual Paulista “Júlio de Mesquita Filho” (FFC/UNESP) approved the study procedures (Approval Number: 4.761.413). All volunteers provided informed consent and were thoroughly informed about the study’s methods and objectives. Additionally, the study was registered with the Clinical Trials Network (clinicaltrials.com, identification code NCT06405477).

Volunteers were recruited through social media and posters displayed throughout the university in Presidente Prudente, São Paulo, Brazil. Those who expressed interest participated in an interview and were asked to complete the IPAQ [23] questionnaire. Participants who met the inclusion criteria were eligible to proceed to the protocol sessions. In this triple-blinded, randomised, placebo-controlled trial, healthy male participants aged 18 to 30, with a body mass index (BMI) ranging from 18.5 to 29.9 kg/m^2^ and who were moderate physically active as determined by the International Physical Activity Questionnaire (IPAQ), were enrolled. Based on the IPAQ, we included volunteers who engaged in vigorous activity for at least 20 min per day on three or more days or in moderate-intensity activity or walking for at least 30 min per day on five or more days.

Individuals who smoked or struggled with alcohol dependence, as well as those exhibiting specific characteristics that could compromise the assessment of autonomic modulation and exercise participation, were excluded from the study. This included individuals with cardiopulmonary, neurological, or metabolic disorders; those with abnormal hemodynamic responses during exercise; and individuals with orthopaedic issues that would hinder their ability to complete the experimental procedure. Furthermore, volunteers diagnosed with COVID-19 within the past 24 weeks, those showing symptoms of the disease, or individuals who had been in contact with confirmed COVID-19 cases in the preceding 14 days were also not included.

### 2.2. Protocols

The participants were instructed to avoid alcoholic beverages and foods containing caffeine for 12 h before each phase of the experimental protocol. They were also advised to have a light meal, drink 500 mL of water two hours before the session, and refrain from intense physical activity the day before. The experimental procedures were organised into two protocols, with a minimum interval of 48 h between them. Before both protocols, body measurements were taken. These sessions took place between 5:00 p.m. and 9:00 p.m. to control for circadian rhythm effects, in a room maintained at a temperature of 23 °C to 26 °C and with humidity levels between 60% and 70% [21]. The two protocols were randomised to reduce the risk of bias.

Following the randomisation process, the participants took six capsules containing either 3 g of starch (placebo protocol) or 3 g of L-arginine (L-arginine protocol). To preserve the blinding of the study, the bottles and their contents were identical in colour and size. Neither the volunteers nor the researchers were aware of the contents of the capsules during the protocols. The order of the protocols was determined using a randomisation process conducted through the “Research Randomizer” website (www.randomizer.org, accessed on 16 February 2024).

Before commencing the experimental procedures, the participants visited the laboratory for body measurements. Their weight was recorded using a digital scale (Welmy W 200/5, Sao Paulo, Brazil), and their height was measured with a stadiometer (ES 2020-Sanny, Sao Paulo, Brazil). Those who met the specified inclusion criteria for the study proceeded to the next research phase.

During the protocols, the volunteers were equipped with a heart rate monitor (Polar RS800CX, Kempele, Finland) to track their heart rates continuously. After the equipment was correctly set up, they were instructed to remain seated for 10 min. Their systolic blood pressure (SBP) and diastolic blood pressure (DBP) were recorded at the end of this period. Following this, the volunteers consumed the substance assigned to them through randomisation and waited for 60 min.

After adhering to the established procedures, the volunteers began their routine with a warm-up on a treadmill, maintaining a speed of 5 km/h for the first 3 min. Following this, the speed was incrementally increased by 1 km/h every 2 min until they reached 80% of their estimated maximum heart rate, calculated using the formula 208 − (0.7 * age). The maximum heart rate projected according to age was determined using the Tanaka formula [24].

Upon completing the exercise, the volunteers remained on the treadmill for 3 min for recovery while being monitored closely.

### 2.3. Outcomes

Heart rate recovery (HRR) was used as an indirect measure to assess the reactivation of the parasympathetic nervous system. To determine HRR, the heart rate (HR) was recorded at four specific intervals: at the end of the exercise (denoted as HRpeak) and at the first (HR1), second (HR2), and third (HR3) minutes of recovery. The values were calculated by taking the arithmetic mean of five consecutive heartbeats, including two beats before and two beats after the HR measurements at HRpeak, HR1, HR2, and HR3.

After recording these values, the differences between HRpeak and HR1, HR2, and HR3 were calculated as follows: HRpeak − HR1 = HRR1; HRpeak − HR2 = HRR2; HRpeak − HR3 = HRR3.

The validated series of RR intervals recorded by the Polar RS800CX heart rate monitor (Polar Electro, Kempele, Finland) was used to analyse the heart rate variability (HRV). The RR interval series underwent digital filtration using the Polar Pro Trainer software (Version 5.0, Polar Inc., Kempele, Finland), with only those sets containing more than 95% sinus beats included in the analysis. Additionally, the RR interval series was visually inspected to identify any artefacts or arrhythmias that could affect the HRV analysis.

In analysing the RMSSD index, we focused on the first two minutes of the recovery period, divided into four 30 s intervals: M1, M2, M3, and M4. This methodology, proposed by Goldberger et al. and utilised in several other studies, is effective in assessing the reactivation of parasympathetic tone following exercise [25].

Systolic blood pressure (SBP) and diastolic blood pressure (DBP) measurements were taken indirectly using a stethoscope (3M Littmann Master Cardiology, Saint Paul, MN, USA) and an aneroid sphygmomanometer (Welch Allyn Tycos, New York, NY, USA) on the participant’s left arm, following a previously described methodology. The pulse pressure (PP) and mean arterial pressure (MAP) were assessed as secondary outcomes.

The pulse pressure (PP) was calculated as the difference between the systolic blood pressure (SBP) and diastolic blood pressure (DBP) using the formula PP = SBP − DBP. The mean arterial pressure (MAP) was then determined by adding one-third of the pulse pressure (PP) to the diastolic blood pressure (DBP) using the formula MAP = (1/3) PP + DBP. For analysis, the baseline values were compared with the measurements taken at the first (1 min) and third (3 min) minutes of recovery.

### 2.4. Data Analysis

Descriptive statistics characterised the sample for data analysis, presenting the results as means, standard deviations, minimum and maximum values, absolute counts, and percentages. The normality of the data was assessed using the Shapiro–Wilk test. A two-way ANOVA with repeated measures was conducted to compare the HRR, root mean square of successive differences (RMSSD30), mean arterial pressure (MAP), and pulse pressure (PP) across the different protocols and time points, as well as the interaction between these factors. Sphericity violations were evaluated using the Mauchly test, and the Greenhouse–Geisser correction was applied when necessary.

Bonferroni’s post hoc test was used for parametric distributions, while Dunn’s post hoc test was used for non-parametric distributions, to analyse moments within the same protocol. The partial eta-squared effect size (P) was calculated for the ANOVA results, with the effect sizes categorised as follows: small effect (≤0.05), medium effect (between 0.06 and 0.13), and large effect (≥0.14). Additionally, Cohen’s d effect size was calculated for comparisons between time points, classified as small effects (<0.50), medium effects (between 0.50 and 0.70), large effects (between 0.80 and 1.20), and very large effects (≥1.30). The level of statistical significance was set at 5%. Analyses were performed using IBM SPSS Statistics, version 22.0 and GraphPad Instat, version 3.01, 1998 (GraphPad Software, Inc., San Diego, CA, USA).

A pilot project involving 13 participants provided the data necessary for the calculation of the sample size using the RMSSD index, which indicated the highest minimum sample size requirement. With a standard deviation of 5.03 ms and a minimum difference of 4.35 ms between the groups, alongside an alpha risk of 0.05 and power of 0.80, the required sample size was determined to be 22 individuals. An additional 10% was included to account for potential dropouts, resulting in a minimum requirement of 24 individuals per group. This calculation used version 3.1.9.7 of GPower (Heinrich-Heine-Universität, Düsseldorf, Germany). A statistical analysis was carried out by a researcher who was unaware of the protocol identities.

## 3. Results

**Figure 1:**
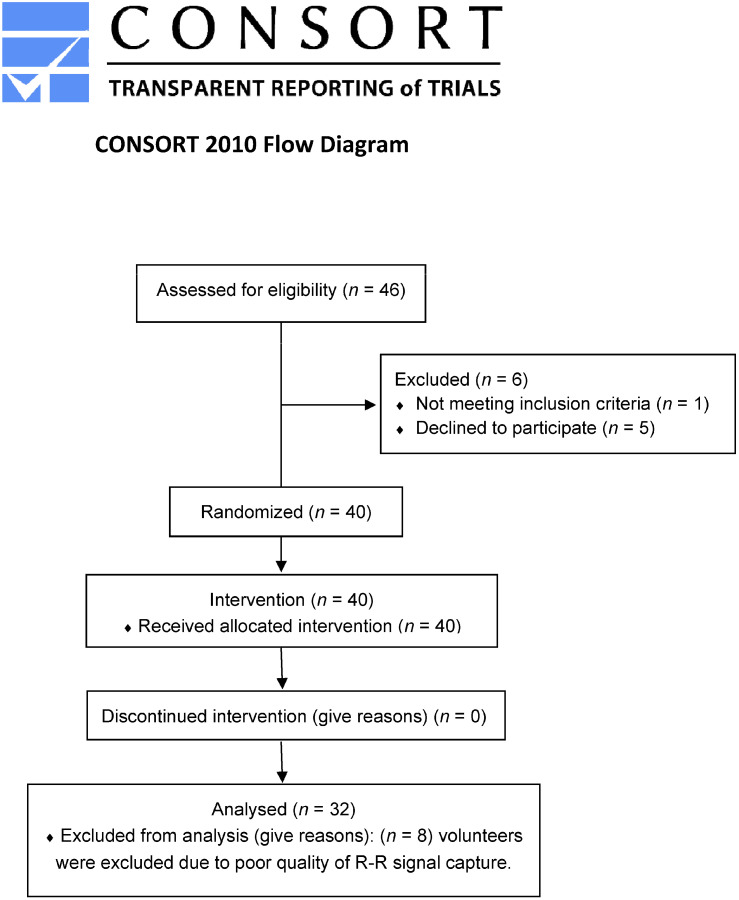
Flowchart diagram.

Table 1 presents the anthropometric characteristics of the 32 participants, while Figure 1 provides a flow diagram illustrating the progress of all participants throughout the study.

**Figure 2:**
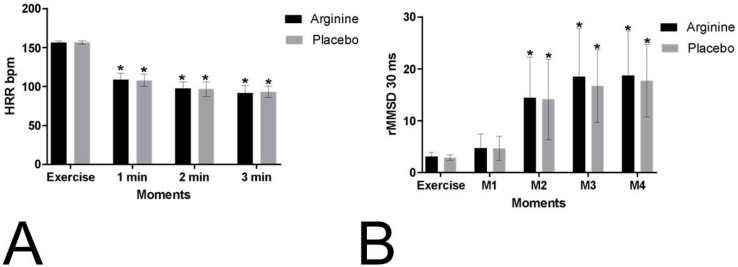
Mean values and their respective standard deviations for heart rate recovery (HRR) (**A**) and root mean square of successive RR interval differences analysed in 30 s intervals (RMSSD30) (**B**) during exercise and recovery obtained from the placebo protocol (placebo) and the L-arginine protocol (arginine) are presented. Caption: 1 min: first minute of recovery; 2 min: second minute of recovery; bpm = beats per minute; M1 = 0–30 s, M2 = 30–60 s, M3 = 60–90 s, M4 = 90–120 s of recovery; ms = milliseconds. * Values indicate significant differences compared to exercise (*p* < 0.05).

The HRR values during the recovery period were compared to those recorded during exercise. No significant differences were observed between the protocols (*p* = 0.944; P = 0.001, power: 0.051) or in the interactions of the moments versus protocols (*p* = 0.530; P = 0.011, power: 1). However, significant differences were identified between the moments themselves (*p* = 0.001; P = 0.964, power: 1), as depicted in Figure 2. The mean HRR values, along with their respective standard deviations, during exercise and recovery at the first and second minutes are presented in Table S1 (Supplementary Materials).

For the RMSSD30 index, no significant differences were found between the protocols (*p* = 0.562; P = 0.005, power: 0.089) or in the interactions of the moments versus protocols (*p* = 0.766; P = 0.005, power: 1). However, significant differences were observed between the moments (*p* = 0.001; P = 0.679, power: 1) (Figure 2). The mean values, along with their standard deviations, of the RMSSD30 index obtained during exercise and immediate recovery are available in Table S2 (Supplementary Materials).

**Figure 3:**
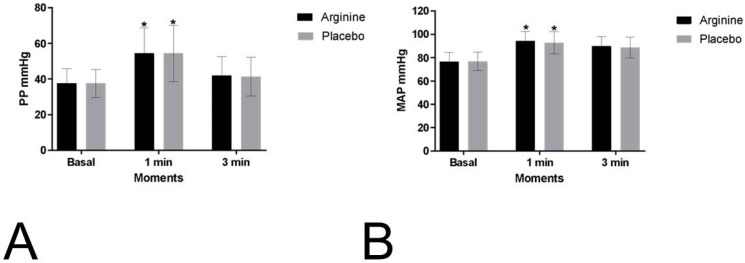
Mean values and their respective standard deviations for pulse pressure (PP) (**A**) and mean arterial pressure (MAP) (**B**) at baseline and during recovery are presented for the placebo protocol (placebo) and the L-arginine protocol (arginine). Caption: 1 min: first minute of recovery; 3 min: third minute of recovery; mmHg = millimetres of mercury. * Values indicate significant differences compared to baseline (*p* < 0.05).

Regarding the secondary outcomes of the mean arterial pressure (MAP) and pulse pressure (PP), no significant differences were observed between the protocols (MAP: *p* = 0.687, P = 0.003, power: 0.068; PP: *p* = 0.929, P = 0.001, power: 0.051) or in the interactions of the moments versus protocols (MAP: *p* = 0.390, P = 0.015, power: 1; PP: *p* = 0.961, P = 0.001, power: 1). However, significant differences were found between the moments for both the MAP (*p* = 0.001, P = 0.825, power: 1) and PP (*p* = 0.001, P = 0.497, power: 1) (Figure 3). The values for these outcomes, recorded during exercise and recovery at the first and third minutes, are provided in Table S3 (Supplementary Materials).

## 4. Discussion

The current study aimed to assess the effects of L-ARG intake on immediate cardiovascular recovery following a submaximal exercise test in healthy adult males. Our findings suggest that the consumption of this substance did not impact immediate parasympathetic reactivation and post-exercise BP recovery.

The decline in HR following exercise is a crucial indicator of autonomic function. A rapid reduction in the post-exercise HR is linked to a favourable cardiovascular prognosis, indicating a healthy autonomic response [26]. Cole et al. [5] defined an abnormal HRR in the first 1 to 3 min as a decrease of 12 beats per minute or less from the peak HR. HRR in the first minute after exercise is considered a predictor of mortality [24], primarily reflecting parasympathetic reactivation [27].

During exercise, the cardiovascular system must adjust to meet the muscles’ metabolic demands and the skin’s thermoregulatory needs while keeping the BP stable. According to the literature, a prominent model suggests that signals from the brain at the onset of exercise prompt the baroreflex to elevate, leading to a swift increase in the heart rate by reducing parasympathetic activity. Muscle mechanoreceptors and increased venous return influence this initial response. As the exercise intensity increases, the parasympathetic activity decreases. In contrast, the sympathetic activity rises, shifting from parasympathetic control at rest to sympathetic control at high intensities, further amplified by sympathoadrenal activation [2].

During physical exercise, HR and BP regulation undergo significant changes. When the cellular metabolism increases, activating the metaboreflex, triggered by the accumulation of metabolic by-products, it reduces the baroreflex function in the brainstem. This activation stimulates nerve fibres, resulting in heightened sympathetic activity and increasing the HR, cardiac output, and BP and vasoconstriction in non-active muscles. Following exercise, these effects are anticipated to diminish rapidly due to the reactivation of parasympathetic activity [28].

Our study examined the physiological behaviour of the heart rate (HR), revealing a significant decrease throughout the recovery period, with a notable reduction occurring in the first minute of passive recovery across all protocols (mean difference in HR during exercise: PLA = 48.85 bpm; L-ARG = 47.41 bpm). In analysing the behaviour of the RMSSD30 index, no significant differences were observed between the protocols, indicating that the ingestion of 3 g of L-ARG did not influence post-exercise parasympathetic reactivation.

Our data are supported by Streeter et al. (2019) [20], who investigated recovery following resistance exercise. The authors [20] conducted the only published study evaluating the effects of L-ARG on autonomic modulation post-exercise and found no influence of this substance on these parameters [20]. This randomised, crossover, double-blind, placebo-controlled clinical trial involved 30 physically active participants (15 men and 15 women). After ingesting either 3 g of L-ARG or 3 g of a placebo, the participants performed five sets of elbow extension–flexion exercises. Significant time effects on autonomic modulation were observed, as indicated by the RMSSD and percentage of successive RR intervals that differed by more than 50 milliseconds (PNN50); however, no treatment or interaction effects were detected. No significant differences were found between the basal values and recovery time for the HR and BP outcomes. Consequently, the study concluded that L-ARG did not affect ANS behaviour.

Despite these similar findings, there are noteworthy methodological differences between the studies. Streeter et al. [20] evaluated a 10 min recovery period without segmenting it to observe and compare the effects over time more effectively, and the recovery values were compared to the basal values. A key difference in the exercise protocol was that it consisted of five sets of 10 maximal isokinetic elbow extensions at 90 degrees per second, with 30 s rests between sets.

We also did not detect significant effects of L-ARG ingestion on post-exercise BP recovery. Across all protocols, this study observed the physiological response of the BP, noting a substantial increase in PP at 1 min of recovery, with the values returning to baseline by 3 min and the MAP not recovering in any protocol. This suggests that, in healthy individuals, endogenous NO production may sufficiently mitigate the effects of 3 g L-ARG consumption [10]. This finding could explain the discrepancy between the results of our clinical trial and those reported in the systematic review by Porto et al. (2021) [29].

In this context, Porto et al. (2021) [29] conducted a systematic review of the effects of L-arginine supplementation on hypertensive adults post-exercise, involving a sample of 60 individuals. Their results indicated a reduction in post-exercise SBP by 5.04 mmHg and DBP by 2.96 mmHg in the L-ARG group compared to the placebo group. It is important to note key differences between these studies. The systematic review included hypertensive men and women over the age of 50. Ageing is directly associated with reduced endothelial activity, increasing the risk of cardiovascular conditions such as hypertension. The decreased availability of NO impairs the body’s ability to maintain endothelial homeostasis [30]. Theoretically, L-arginine, the primary substrate for NO production, could help to address this deficiency [10].

Our study highlights several critical methodological points that warrant attention. Notably, analyses of the plasma nitrate and nitrite concentrations were not conducted, and the low dose of L-ARG may have been insufficient to elicit the expected positive effects of this protocol. A significant limitation of our study was the absence of a standardised ergonomic assessment to measure the cardiopulmonary capacity, specifically VO₂ max, which impacted the precise characterisation of the physical capacity levels of our volunteers. However, our sample size calculation indicated that the number of volunteers included was adequate. The selection process adhered to stringent exclusion criteria, enhancing the reliability of our data. We advise caution in generalising our findings to other groups and recommend that future research involve diverse populations, various doses, and extended treatment durations.

Furthermore, individuals show variability in autonomic recovery post-exercise, particularly in their heart rate recovery (HRR) and blood pressure (BP) responses. While some people return to near-baseline levels within 1–3 min, others may require more time due to intrinsic factors [30]. Recognising this individual variability is crucial in interpreting autonomic recovery results, as quicker recovery generally suggests better cardiovascular and autonomic function. In contrast, slower recovery may require closer monitoring or training adjustments [31].

## 5. Conclusions

L-ARG supplementation did not significantly change immediate post-exercise autonomic recovery among healthy men.

## Figures and Tables

**Table 1:** Characteristics of participants (*n* = 32).

Variable	Mean ± SD	Minimum–Maximum
Age (years)	22.13 ± 2.79	[18–29]
Height (m)	176.45 ± 7.04	[1.65–1.93]
Weight (kg)	77.72 ± 12.47	[57.40–106.60]
BMI (kg/m^2^)	24.73 ± 3.26	[19.50–29.80]

SD = standard deviation; kg = kilogram; m = metres; BMI = body mass index.

## Data Availability

The original contributions presented in the study are included in the article/supplementary materials, further inquiries can be directed to the corresponding author.

## Supplementary Materials

The following supporting information can be downloaded at: https://www.mdpi.com/article/10.3390/nu16234067/s1, Table S1. The behaviour of HRR during the protocols; Table S2. The behaviour of RMSSD_30_ during the protocols; Table S3. The behaviour of PP and MAP during the protocols.

## References

[1] Pastre C.M., Bastos F.D.N., Júnior J.N., Vanderlei L.C.M., Hoshi R.A. (2009). Post-exercise recovery methods: A systematic review. Rev. Bras. Med. Esporte.

[2] Freeman J.V., Dewey F.E., Hadley D.M., Myers J., Froelicher V.F. (2006). Autonomic Nervous System Interaction with the Cardiovascular System During Exercise. Prog. Cardiovasc. Dis..

[3] Peçanha T., Silva-Júnior N.D., Forjaz C.L.d.M. (2014). Heart rate recovery: Autonomic determinants, methods of assessment and association with mortality and cardiovascular diseases. Clin. Physiol. Funct. Imaging.

[4] Vanderlei L.C.M., Pastre C.M., Hoshi R.A., de Carvalho T.D., de Godoy M.F. (2009). Basic notions of heart rate variability and its clinical applicability. Rev. Bras. Cir. Cardiovasc..

[5] Cole C.R., Foody J.M., Blackstone E.H., Lauer M.S. (2000). Heart rate recovery after submaximal exercise testing as a predictor of mortality in a cardiovascularly healthy cohort. Ann. Intern. Med..

[6] Gilmartin J., Bath-Hextall F., Maclean J., Stanton W., Soldin M. (2016). Quality of life among adults following bariatric and body contouring surgery: A systematic review. JBI Database Syst. Rev. Implement. Rep..

[7] Aydin M., Kose E., Odabas I., Meric Bingul B., Demirci D., Aydin Z. (2021). The Effect of Exercise on Life Quality and Depression Levels of Breast Cancer Patients. Asian Pac. J. Cancer Prev..

[8] D’angelo J., Ritchie S.D., Oddson B., Gagnon D.D., Mrozewski T., Little J., Nault S. (2023). Using Heart Rate Variability Methods for Health-Related Outcomes in Outdoor Contexts: A Scoping Review of Empirical Studies. Int. J. Environ. Res. Public Health.

[9] Kloter E., Barrueto K., Klein S.D., Scholkmann F., Wolf U. (2018). Heart Rate Variability as a Prognostic Factor for Cancer Survival—A Systematic Review. Front. Physiol..

[10] Mazzeo A.T., LA Monaca E., DI Leo R., Vita G., Santamaria L.B. (2011). Heart rate variability: A diagnostic and prognostic tool in anesthesia and intensive care. Acta Anaesthesiol. Scand..

[11] de Andrade P.E., Amaral J.A.T.D., Paiva L.d.S., Adami F., Raimudo J.Z., Valenti V.E., de Abreu L.C., Raimundo R.D. (2017). Reduction of heart rate variability in hypertensive elderly. Blood Press..

[12] Sassi R., Cerutti S., Lombardi F., Malik M., Huikuri H.V., Peng C.-K., Schmidt G., Yamamoto Y., Reviewers D., Gorenek B. (2015). Advances in heart rate variability signal analysis: Joint position statement by the e-Cardiology ESC Working Group and the European Heart Rhythm Association co-endorsed by the Asia Pacific Heart Rhythm Society. Europace.

[13] Porto A.A., Benjamim C.J.R., Sobrinho A.C.d.S., Gomes R.L., Gonzaga L.A., Rodrigues G.d.S., Vanderlei L.C.M., Garner D.M., Valenti V.E. (2023). Influence of Fluid Ingestion on Heart Rate, Cardiac Autonomic Modulation and Blood Pressure in Response to Physical Exercise: A Systematic Review with Meta-Analysis and Meta-Regression. Nutrients.

[14] O’connor E., Mündel T., Barnes M.J. (2022). Nutritional Compounds to Improve Post-Exercise Recovery. Nutrients.

[15] Álvares T.S., Meirelles C.M., Bhambhani Y.N., Paschoalin V.M.F., Gomes P.S.C. (2011). L-arginine as a potential ergogenic aid in healthy subjects. Sports Med..

[16] Tang J.E., Lysecki P.J., Manolakos J.J., MacDonald M.J., Tarnopolsky M.A., Phillips S.M. (2011). Bolus Arginine Supplementation Affects neither Muscle Blood Flow nor Muscle Protein Synthesis in Young Men at Rest or After Resistance Exercise. J. Nutr..

[17] Gambardella J., Khondkar W., Morelli M.B., Wang X., Santulli G., Trimarco V. (2020). Arginine and Endothelial Function. Biomedicines.

[18] Shiraseb F., Asbaghi O., Bagheri R., Wong A., Figueroa A., Mirzaei K. (2022). Effect of l-Arginine Supplementation on Blood Pressure in Adults: A Systematic Review and Dose–Response Meta-analysis of Randomized Clinical Trials. Adv. Nutr. Int. Rev. J..

[19] Chowdhary S., Vaile J.C., Fletcher J., Ross H.F., Coote J.H., Townend J.N. (2000). Nitric Oxide and Cardiac Autonomic Control in Humans. Hypertension.

[20] Streeter D.M., Trautman K.A., Bennett T.W., McIntosh L.E., Grier J.W., Stastny S.N., Hackney K.J. (2019). Endothelial, Cardiovascular, and Performance Responses to L-Arginine Intake and Resistance Exercise. Int. J. Exerc. Sci..

[21] Pardini R., Matsudo S., Araújo T., Matsudo V., Andrade E., Braggion G., Andrade D., Oliveira L., Figueira A., Raso V. (2001). Validação do questionário internacional de nível de atividade física (IPAQ-versão 6): Estudo piloto em adultos jovens brasileiros Validation. Rev. Bras. Ciência Mov..

[22] Devrim-Lanpir A., Ihász F., Demcsik M., Horváth A.C., Góczán P., Czepek P., Takács J., Kimble R., Zare R., Gunes F.E. (2024). Effects of Acute Citrulline Malate Supplementation on CrossFit^®^ Exercise Performance: A Randomized, Double-Blind, Placebo-Controlled, Cross-Over Study. Nutrients.

[23] Li P., Sun S., Zhang W., Ouyang W., Li X., Yang K. (2024). The Effects of *L*-citrulline Supplementation on the Athletic Performance, Physiological and Biochemical Parameters, Antioxidant Capacity, and Blood Amino Acid and Polyamine Levels in Speed-Racing *Yili* Horses. Animals.

[24] Catai A.M., Pastre C.M., de Godoy M.F., da Silva E., Takahashi A.C.d.M., Vanderlei L.C.M. (2020). Heart rate variability: Are you using it properly? Standardisation checklist of procedures. Braz. J. Phys. Ther..

[25] Tanaka H., Monahan K.D., Seals D.R. (2001). Age-Predicted Maximal Heart Rate Revisited. J. Am. Coll. Cardiol..

[26] Barbosa M.P.d.C.d.R., da Silva N.T., de Azevedo F.M., Pastre C.M., Vanderlei L.C.M. (2016). Comparison of Polar^®^RS800G3™ heart rate monitor with Polar^®^ S810i™ and electrocardiogram to obtain the series of RR intervals and analysis of heart rate variability at rest. Clin. Physiol. Funct. Imaging.

[27] Cole C.R., Blackstone E.H., Pashkow F.J., Snader C.E., Lauer M.S. (1999). Heart-Rate Recovery Immediately after Exercise as a Predictor of Mortality. N. Engl. J. Med..

[28] Savin W.M., Davidson D.M., Haskell W.L., Best S.A., Bivens T.B., Palmer M.D., Boyd K.N., Galbreath M.M., Okada Y., Carrick-Ranson G. (1982). Autonomic contribution to heart rate recovery from exercise in humans. J. Appl. Physiol. Respir. Environ. Exerc. Physiol..

[29] Porto A.A., Gonzaga L.A., Benjamim C., Garner D., Adami F., Valenti V. (2022). Effect of oral l-arginine supplementation on post-exercise blood pressure in hypertensive adults: A systematic review with meta-analysis of randomized double-blind, placebo-controlled studies. Sci. Sports.

[30] Michael S., Graham K.S., Davis G.M. (2017). Cardiac autonomic responses during exercise and post-exercise recovery using heart rate variability and systolic time intervals—A review. Front. Physiol..

[31] van de Vegte Y.J., Tegegne B.S., Verweij N., Snieder H., van der Harst P. (2019). Genetics and the heart rate response to exercise. Cell. Mol. Life Sci..

