# Supplementary material for: L-Arginine Supplementation Did Not Impact the Rapid Recovery of Cardiovascular and Autonomic Function Following Exercise in Physically Active Healthy Males: A Triple-Blind Randomised Placebo-Controlled Crossover Trial
Source: Nutrients. 2024 Nov 27;16(23):4067. doi: 10.3390/nu16234067 (PMC11643886; doi:10.3390/nu16234067)
Supplement: Supplementary file 1 [file nutrients-16-04067-s001.zip › nutrients-3298276-supplementary.pdf]

Table S1. The behaviour of HRR during the protocols.

Variable	Protocol	Exercise	1 Min	2 Min	3 Min	Cohen d′
**HRR bpm**	L-ARG	156.53 ± 2.27 (157.34–155.71)	**109.12 ± 7.90 (111.97–106.27) [47,41]**	**97.62 ± 8.17 (100.56–94.67) [58,91]**	**91.68 ± 9.70 (95.17–88.18) [64,84]**	1 min = 8.16<br>2 min = 9.83<br>3 min = 9.21
	PLA	156.74 ± 2.24 (157.51–155.96)	**107.89 ± 7.89 (110.63–105.16) [48,85]**	**96.74 ± 9.68 (100.09–93.39) [60]**	**93.17 ± 7.28 (95.69–90.65) [63,57]**	1 min = 8.42<br>2 min = 8.54<br>3 min = 11.80

Mean ± standard deviation; (95% confidence interval); [difference relative to exercise]. Cohen d′ = exercise vs. post-exercise recovery (1 min, 2 min and 3 min). Caption = 1 min = first minute of recovery; 2 min = second minute of recovery; HRR = heart rate recovery; bpm = beats per minute; L-ARG = L-arginine protocol; PLA = Placebo protocol. Values in **bold** = significant difference between exercise and rest moments ($p < 0.05$), two-way ANOVA for repeated measures followed by Bonferroni post-test.

Table S2. The behaviour of $RMSSD_{30}$ during the protocols.

Indice	Protocol	Exercise	Moments				Cohen d′
			M1	M2	M3	M4	
$RMSSD_{30}$ ms	L-ARG	3.09 ± 0.86 (3.39–2.79)	4.71 ± 2.72 (5.66–3.77)	**14.41 ± 7.87 (17.14–11.68)**	**18.51 ± 9.35 (21.75–15.27)**	**18.71 ± 8.59 (21.68–15.73)**	M1 = 0.80<br>M2 = 2.02<br>M3 = 2.32<br>M4 = 2.56
	PLA	2.88 ± 0.54 (3.07–2.70)	4.69 ± 2.34 (5.50–3.88)	**14.11 ± 7.77 (16.80–11.42)**	**16.70 ± 7.00 (19.12–14.28)**	**17.74 ± 7.00 (20.17–15.31)**	M1 = 1.07<br>M2 = 2.04<br>M3 = 2.78<br>M4 = 2.99

Mean ± standard deviation; (95% confidence interval); Cohen d′ = exercise vs. post-exercise recovery (M1, M2, M3 and M4). Caption = M1 = 0–30 seconds (s), M2 = 30–60 s, M3 = 60–90 s, M4 = 90–120 s of recovery; $RMSSD_{30}$ = root mean square of successive RR interval differences analyzed in 30-s intervals; ms = milliseconds; L-ARG = L-arginine protocol; PLA = placebo protocol. Values in **bold** = significant difference between the moments of exercise and rest ($p < 0.05$), two-way ANOVA for repeated measures followed by Bonferroni or Dunn post-test.

Table S3. The behaviour of PP and MAP during the protocols.

Variables	Protocol	Moments			
		Basal	1 Min	3 Min	Cohen d′
**PP mmHg**	L-ARG	37.50 ± 8.29 (59.67–15.32)	**54.37 ± 14.34 (71.13–37.61)**	41.87 ± 10.73 (61.70–22.04)	1 min = 1.44 3 min = 0.46
	PLA	37.50 ± 7.90 (61.20–13.79)	**54.37 ± 15.79 (70.56–38.18)**	41.25 ± 10.82 (61.08–21.41)	1 min = 1.34 3 min = 0.39
**MAP mmHg**	L-ARG	76.56 ± 7.97 (100.26–52.85)	**94.37 ± 8.22 (116.54–72.20)**	**89.89 ± 8.18 (100.26–52.85)**	1 min = 2.20 3 min = 1.65
	PLA	76.87 ± 7.85 (100.58–53.16)	**92.81 ± 9.43 (113.71–71.90)**	**88.75 ± 8.80 (110.92–66.57)**	1 min = 1.84 3 min = 1.42

Mean ± standard deviation; (95% confidence interval); Cohen d′ = Basal vs. recovery (1 min and 3 min). Caption: PP: pulse pressure; MAP: mean arterial pressure; L-ARG: L-arginine protocol; PLA: placebo protocol; mmHg: millimeters of mercury; min: minute. Values in **bold**: significant difference between the moments of recovery and exercise ($p < 0.05$). [Two-way ANOVA for repeated measures followed by Bonferroni or Dunn post-test].
